# The promises and perils of circulating tumor DNA for monitoring immunotherapy response in non-small cell lung cancer

**DOI:** 10.37349/etat.2024.00280

**Published:** 2024-11-26

**Authors:** Brandon Joseph Hebert, James Bradley

**Affiliations:** IRCCS Istituto Romagnolo per lo Studio dei Tumori (IRST) “Dino Amadori”, Italy; ^1^Division of General Internal Medicine, Department of Medicine, University of Louisville School of Medicine, Louisville, KY 40202, USA; ^2^Division of Pulmonary, Critical Care, and Sleep Disorders Medicine, Department of Medicine, University of Louisville School of Medicine, Louisville, KY 40202, USA

**Keywords:** Circulating tumor DNA, lung cancer, cancer, liquid biopsy, non-small cell lung cancer

## Abstract

There has been a rapid expansion of immunotherapy options for non-small cell lung cancer (NSCLC) over the past two decades, particularly with the advent of immune checkpoint inhibitors. Despite the emerging role of immunotherapy in adjuvant and neoadjuvant settings though, relatively few patients will respond to immunotherapy which can be problematic due to expense and toxicity; thus, the development of biomarkers capable of predicting immunotherapeutic response is imperative. Due to the promise of a noninvasive, personalized approach capable of providing comprehensive, real-time monitoring of tumor heterogeneity and evolution, there has been wide interest in the concept of using circulating tumor DNA (ctDNA) to predict treatment response. Although the use of ctDNA to detect actionable mutations such as *EGFR* is now integral in the standard of care for patients with NSCLC, several large studies have also shown its potential as a biomarker of immunotherapeutic response. Ongoing ctDNA interventional clinical trials, such as the BR.36 trial, will help to clarify the potential role of ctDNA for therapeutic guidance. Despite the promise of this technology, there are many limitations and considerations that clinicians need to be aware of prior to widespread implementation in clinical practice, such as the effect of underlying comorbidities, ctDNA fraction, stage of underlying malignancy, and concordance between aberrations detected in ctDNA and tumor tissue.

## Introduction

Non-small cell lung cancer (NSCLC) represents a substantial global health burden, exhibiting high morbidity and mortality in countries around the world. Even in developed countries with extensive resources, NSCLC remains challenging to treat. Although conventional therapies such as surgery, chemotherapy, and radiation have been used for decades, mortality rates remain abysmal. However, the emergence of immunotherapeutic strategies has drastically altered the treatment paradigm for a subset of patients with NSCLC. Compared to the historical 5-year survival rate of approximately 5%, multiple studies have shown that immunotherapy can achieve an overall survival rate of > 15% and there is now increasing interest in the use of combination immunotherapy which may further improve the survival rate [1, 2].

Despite the promise of immunotherapy though, only 20–25% of NSCLC patients respond to treatment [3]. Although controversial, some studies suggest the variable response rate to specific immunotherapies may be impacted by programmed death-ligand 1 (PD-L1) expression or tumor mutation burden, establishing a clear need for predictive biomarkers of immunotherapy response [4, 5]. Furthermore, a growing body of literature suggests that the cost-effectiveness of immunotherapy varies widely and may be prohibitive without the use of biomarkers to guide patient selection [6]. Although tissue biopsy is the most common method for NSCLC identification and molecular profiling, invasive sampling often fails to reflect tumor heterogeneity, reflecting the need for the development of non-invasive testing strategies capable of monitoring the molecular evolution of tumors over time and in response to treatment [7].

**Figure 1:**
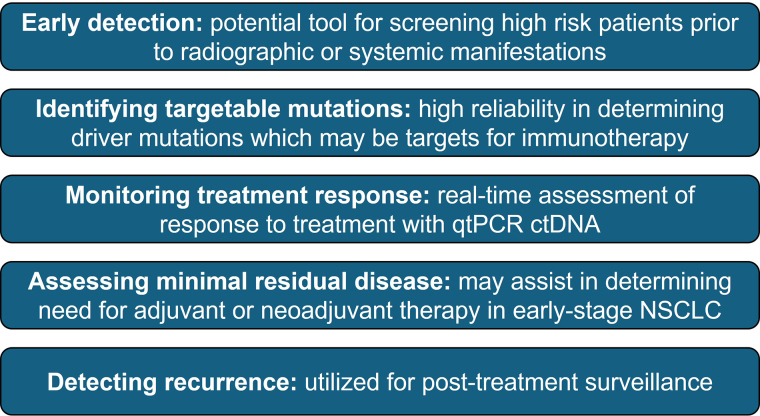
Potential use of ctDNA in NSCLC. qtPCR: quantitative real-time polymerase chain reaction; ctDNA: circulating tumor DNA; NSCLC: non-small cell lung cancer

Over the past decade, one of the most promising biomarkers that have been proposed is circulating tumor DNA (ctDNA). In 2016, the FDA approved the first clinical application of ctDNA for the detection of *EGFR* mutations. Due to the improvement in speed, accuracy, and cost of next-generation sequencing technologies, several commercial platforms have been developed that rely on the detection of ctDNA. In the era of immunotherapy, multiple studies have shown that detection of ctDNA has the potential to aid in the management of patients with NSCLC, and it is now considered an integral part of routine clinical practice (Figure 1).

Although initially used to detect *EGFR* mutations in NSCLC, studies have shown that ctDNA may also be used for the detection of other targetable and non-targetable driver mutations, such as *KRAS*, *PIK3CA*, *TP53*, and *MET*. Additionally, ctDNA has been proposed as an adjunct for screening high-risk individuals, primary diagnosis, treatment monitoring, evaluation of minimal residual disease, and post-treatment surveillance. Interestingly, emerging evidence suggests that ctDNA may have prognostic significance when used to determine the response to immunotherapy. Goldberg et al. [8] found that a > 50% decrease in ctDNA levels from baseline was strongly correlated with radiographic response and was associated with longer progression-free survival and overall survival in patients with metastatic NSCLC receiving immune checkpoint inhibitors. Subsequent studies by Anagnostou et al. [9] and Raja et al. [10] found that patients with a reduction in ctDNA levels after initiating therapy had better clinical outcomes compared to non-responders. Similarly, a recent meta-analysis of 522 patients with NSCLC found that reduction of ctDNA 6–16 weeks after treatment was associated with improved progression-free survival and overall survival [11].

Identifying predictive biomarkers of benefit from immunotherapy is challenging because immunotherapy biomarkers are continuous, temporally variable, and influenced by multiple interactions as opposed to oncogenic biomarkers which are binary and fixed (the mutation is either present or not present). Over the past decade, there have been an increasing number of ctDNA-directed clinical trials which will help solidify the role of ctDNA analysis in precision immuno-oncology, however prior to implementation in routine clinical practice, it is important for clinicians to understand the limitations and promise of ctDNA-based technology when assessing the ability to predict immunotherapy response (Table 1).

**Table 1:** Benefits and limitations of ctDNA-based technology for the assessment of immunotherapy response

**Benefits**	**Limitations**
Reduction in ctDNA levels associated with overall survival and progression-free survival	Comorbidities can affect ctDNA levels (smoking, pregnancy, heart disease, inflammation)
Detection of immunotherapy response after about 8 weeks	Clonal hematopoiesis
Allows for serial monitoring to detect developing resistance and genomic variants	Variable ctDNA fraction based on primary malignancy and stage
Non-invasive detection (blood test)	Multiple assays to choose from with no standardization
Comprehensive, patient-specific molecular profiling	Wide variability in cost
-	Concordance between aberrations detected in ctDNA and tumor tissue
-	Low concentration and fragmentation
-	Unable to detect genomic fusion events and copy number changes

ctDNA: circulating tumor DNA; -: no data

## Promises for predicting immunotherapy response

Although many cancer biomarkers have been investigated in the literature, most of them fail to become clinically useful because they are not helpful (the test does not provide an actionable result) or the positive and negative predictive values of the test are suboptimal for clinical decision-making in real-world scenarios. ctDNA is increasingly utilized in the management of NSCLC due to its ability to provide real-time insights into tumor dynamics through a minimally invasive approach and is a valuable tool for actionable, targeted, personalized cancer care. As described in further detail elsewhere, the applications of ctDNA for the diagnosis, prognostication, and monitoring of NSCLC patients are actively being investigated by many groups [12]. Several studies have recently been performed which highlight the optimism and promise for ctDNA assessment to predict immunotherapy response.

A systematic review by Wang et al. [13], involving 1,017 patients and 10 studies, highlighted that early reduction of ctDNA in patients with NSCLC who received immunotherapy was associated with improvement in progression-free survival, overall survival, and objective response rate. Subsequently, a pooled analysis of five clinical trials revealed a strong association between reductions in ctDNA and improvement in overall survival (hazard ratio, 2.28; 95% CI, 1.62 to 3.20; *P* < 0.001), progression-free survival (hazard ratio 1.76; 95% CI, 1.31 to 2.36; *P* < 0.001), and tumor response [14].

The purpose of an ongoing multi-center, randomized, ctDNA-directed phase two trial (BR.36) is to establish the role of ctDNA as a potential early biomarker of immunotherapy response. In this trial, results showed that immunotherapy response could be detected within about 8 weeks following treatment initiation with immunotherapy. In the first stage of the trial, results showed a sensitivity of ctDNA response for Response Evaluation Criteria in Solid Tumors (RECIST) response of 82% with a specificity of 75% [15]. During the second stage of this trial, which is currently recruiting patients, patients at risk of progression will be randomized to continuation of therapy versus treatment intensification.

Data support elective immunotherapy discontinuation after two years and a proof-of-concept study showed that sustained ctDNA clearance can identify patients appropriate for immunotherapy de-escalation [16]. Similarly, a small non-randomized controlled trial involving patients with advanced NSCLC showed that it was feasible to use ctDNA as part of an adaptive de-escalation treatment strategy to identify patients who achieve complete remission after consolidative therapy [17]. In *EGFR*-mutant NSCLC, the APPLE trial demonstrated the feasibility of serial monitoring of ctDNA to inform treatment decisions, identifying 17% of patients with molecular progression prior to RECIST progression [18].

## Limitations in the use of ctDNA in NSCLC

Despite the potential of ctDNA to revolutionize patient selection for immunotherapy, there are several important limitations that clinicians must be aware of when implementing this technology in practice. There are a variety of factors that can falsely elevate ctDNA levels and impact the accuracy of ctDNA analysis. While some are intrinsic to the technology, extrinsic factors, such as patient comorbidities, variable sample preparation protocols, and tumor characteristics are known to influence ctDNA. For example, ctDNA levels may be influenced by smoking, pregnancy, heart disease, inflammation, physical activity, and even diurnal variations. Additionally, rewarming samples for more than three days at 40°C may result in falsely elevated levels of ctDNA [19]. Understanding and acknowledging the impact of these limitations will assist clinicians in determining the optimal strategy when using ctDNA analysis in their clinical practice and individual programs.

Multiple cells, malignant and non-malignant, secrete cell-free DNA (cfDNA) through a variety of mechanisms. The majority of cfDNA is released by hematopoietic cells, and in 10–20% of patients over the age of 70, these cells can acquire mutations that establish a non-malignant clonal population of cells. Some of these mutations occur in genes such as *EGFR* and *TP53*, which may complicate the clinical interpretation of ctDNA in older patients [20]. Additionally, cfDNA is released into the blood via multiple mechanisms including apoptosis, necrosis, and active secretion which may affect the interpretation of ctDNA in NSCLC. However, there have been studies of cfDNA in NSCLC which have shown a decreased ctDNA/cfDNA ratio is associated with better prognosis [21]. Additionally, clinicians need to be aware that the ctDNA fraction (proportion of tumor-derived cfDNA), which can range from 0.01–90%, can be impacted by the tumor’s size, location, and vascularity. Variable fractions of ctDNA result in fluctuating sensitivity for detecting genomic alterations (i.e., higher amounts of ctDNA correlate with higher concordance with biopsy specimens); additionally, ctDNA fraction varies between different primary malignancies [22]. A systematic review found that the use of ctDNA for screening for early cancer may be problematic as there was little utility in tumors < 1 cm in size [19].

Although several commercial assays have now been developed to detect ctDNA in lung cancer patients, there exists wide variability in the sensitivity and specificity between assays which could affect the ability of clinicians to accurately assess treatment response to immunotherapy [19]. Additionally, it is increasingly recognized that early-stage tumors release very low amounts of ctDNA as opposed to late-stage or disseminated disease, which could subsequently result in a higher number of false negative results in patients with early-stage disease [23]. Although approximately 75% of NSCLC patients have stage III/IV disease at the time of diagnosis, there has been an increasing number of patients diagnosed with early-stage lung cancer over the past two decades [24]. For example, CancerSEEK utilizes a combination of ctDNA and eight different proteins to screen for multiple types of cancer of the cancers that were assessed, it was notably least accurate in patients with lung cancer and in patients with stage I disease [25]. As immunotherapy is increasingly being used in the treatment of early-stage NSCLC, both in the neoadjuvant and adjuvant settings, the limitations of detecting ctDNA at early stages of the disease will have a subsequent impact on the tests’ ability to predict immunotherapy response in this patient population. This suggests that utilizing ctDNA to assess immunotherapy response may exhibit variable performance based on stage at the time of diagnosis, with superior predictive ability in patients with late-stage disease.

With the dramatic increase in utilization of PCR-based and next-generation sequencing-based technologies, the development of multiple platforms (and no universal standardization) has led to wide variability in costs. Kramer et al. [26] estimated that costs can vary from $199–$9,124 depending on the assay, setting, and specimen volume. Given the significant financial burden associated with treating late-stage NSCLC as well as immunotherapy costs, it will be imperative to use platforms that are cost-effective for patient care when utilizing ctDNA to assess immunotherapy response. This may negatively impact the use of ctDNA in patients from a low socioeconomic background, further exacerbating existing healthcare disparities. Encouragingly, a recent systematic review by Fagery et al. [27] found that the detection of ctDNA is potentially cost-effective in the selection of treatment for patients with lung cancer, however, no studies have addressed the cost-benefit of this technology when used for immunotherapy response prediction.

The concordance between aberrations detected in ctDNA and tumor tissue is an additional limitation that may impact the ability to monitor immunotherapy response. In patients with NSCLC, a recent report by Tran et al. [28] found an 80% concordance rate between ctDNA and tissue biopsy at the time of diagnosis, however, concordance dropped to 40% at the time of progression. While ctDNA shows high concordance with tissue biopsy for many genetic alterations, some discrepancies exist. For instance, ctDNA may detect subclonal drivers of resistance not captured in tissue sequencing, which can be crucial for guiding therapy decisions. However, certain alterations, such as gene amplifications, maybe less frequently detected in ctDNA compared to tissue biopsy. Regardless of the ctDNA platform that is used, discordance between ctDNA and tumor tissue may be attributed to intratumoral heterogeneity, clonal evolution, sampling bias, and time-lapse from sample acquisition. It is currently unknown how the discordance rate between ctDNA and tumor samples would impact immunotherapy response prediction models.

An additional limitation of the use of ctDNA to monitor immunotherapy response is the presence of concurrent therapeutic regimens. In a small study by Murray et al. [29], which examined the longitudinal ctDNA dynamics following initiation of immunotherapy for NSCLC, the authors found that for patients receiving single-agent immunotherapy, there was a difference in progression-free survival which was dependent on the frequency of mutant alleles detected via ctDNA. In contrast, for patients receiving chemoimmunotherapy, there was no correlation between mutant allele frequencies and outcomes. This suggests the possibility that concurrent treatment regimens may impact the ability to use ctDNA to monitor immunotherapy response.

## Future directions

In the future, response assessment to immunotherapy will likely require a multi-parameter approach, which may include the combination of immune cell profiling, radiomics, and ctDNA dynamics, to accurately predict which patients may benefit from immunotherapeutic agents, the optimal duration of therapy, and post-treatment surveillance for recurrence and development of resistant mutations. The complexity of the immune response to tumors is not fully understood with ctDNA analysis, reinforcing the need for future studies that seek to understand the immune compartment and tumor microenvironment interactions.

Further investigation and consensus on a definition of “ctDNA-based molecular response” that clinically describes a patient population receiving benefit from immunotherapy is needed as there is significant heterogeneity in the current published literature. While several studies have clearly shown that a decrease in ctDNA following treatment is associated with improvement in progression-free survival and overall survival, other studies defined ctDNA clearance (or ctDNA level reduction below the limit of detection) as the predictive biomarker of response. In 2017, Chaudhuri et al. [30] showed that patients with detectable ctDNA at any post-treatment point had significantly decreased freedom from progression and overall survival than those with undetectable ctDNA. Detection of ctDNA was strongly prognostic in this study regardless of treatment received (stereotactic ablation radiotherapy versus surgery versus chemoradiotherapy). Similarly, for NSCLC patients receiving treatment with epidermal growth factor tyrosine kinase inhibitors, early undetectable ctDNA showed a statistically significant improvement in progression-free survival and overall survival compared to patients with detectable ctDNA [21].

Additionally, further evaluation of ctDNA kinetics following immunotherapy is needed to help better define the optimal duration of time after treatment at which ctDNA assessment may serve as an endpoint of response. Within the current National Comprehensive Cancer Network (NCCN) Clinical Practice Guidelines in Oncology for NSCLC, monitoring is only recommended after 2 cycles of systemic chemotherapy and then every 2 to 4 cycles with computed tomography (CT) chest, abdomen, and pelvis [31]. Some studies have shown that detection of ctDNA preceded radiographic evidence of disease by up to 5 months; thus, detection of ctDNA in the future may be an essential resource in monitoring disease response to therapy. Several ctDNA interventional clinical trials for patients with advanced NSCLC receiving immunotherapy are currently ongoing as they seek to further investigate the utility of ctDNA for therapeutic guidance, and as more data becomes available, it may be imperative to more strictly define timeframes for disease monitoring following treatment for NSCLC.

## Abbreviations

cfDNA: cell-free DNA
ctDNA: circulating tumor DNA
NSCLC: non-small cell lung cancer
RECIST: Response Evaluation Criteria in Solid Tumors

## Declarations

### Copyright

© The Author(s) 2024.
